# Anti- and pro-fibrillatory effects of pulmonary vein isolation gaps in human atrial fibrillation digital twins

**DOI:** 10.1038/s41746-024-01075-y

**Published:** 2024-03-26

**Authors:** Ze Jin, Taehyun Hwang, Daehoon Kim, Byounghyun Lim, Oh-Seok Kwon, Sangbin Kim, Moon-Hyun Kim, Je-Wook Park, Hee Tae Yu, Tae-Hoon Kim, Jae-Sun Uhm, Boyoung Joung, Moon-Hyoung Lee, Hui-Nam Pak

**Affiliations:** 1 Division of Cardiology, Department of Internal Medicine, Yonsei University College of Medicine, Seoul, Republic of Korea; 2 Division of Cardiology, Department of Internal Medicine, Yongin Severance Hospital, Yonsei University College of Medicine, Yongin, Republic of Korea

**Keywords:** Atrial fibrillation, Computational models

## Abstract

Although pulmonary vein isolation (PVI) gaps and extrapulmonary vein triggers contribute to recurrence after atrial fibrillation (AF) ablation, their precise mechanisms remain unproven. Our study assessed the impact of PVI gaps on rhythm outcomes using a human AF digital twin. We included 50 patients (76.0% with persistent AF) who underwent catheter ablation with a realistic AF digital twin by integrating computed tomography and electroanatomical mapping. We evaluated the final rhythm status, including AF and atrial tachycardia (AT), across 600 AF episodes, considering factors including PVI level, PVI gap number, and pacing locations. Our findings revealed that antral PVI had a significantly lower ratio of AF at the final rhythm (28% vs. 56%, *p* = 0.002) than ostial PVI. Increasing PVI gap numbers correlated with an increased ratio of AF at the final rhythm (*p* < 0.001). Extra-PV induction yielded a higher ratio of AF at the final rhythm than internal PV induction (77.5% vs. 59.0%, *p* < 0.001). In conclusion, our human AF digital twin model helped assess AF maintenance mechanisms. Clinical trial registration: https://www.clinicaltrials.gov; Unique identifier: NCT02138695.

## Introduction

Circumferential pulmonary vein (PV) isolation (PVI) is an essential procedure for atrial fibrillation (AF) catheter ablation (AFCA) and a class I indication for treating antiarrhythmic drug (AAD)-resistant AF according to the guidelines^1^. However, a PVI at the PV ostial level and antral-wide circumferential PVI differ regarding atrial mass reduction. Balloon technologies, such as cryoballoon PVI, differ from conventional radiofrequency (RF) PVI based on the balloon–tissue contact surface and the shape of the lesion^2^. PV reconnections, which can occur even after an acute success of a PVI, are a major AF recurrence factor^3^. In the second look-mapping studies conducted 3 months post-procedure, PV reconnection rates per patient were 65%^4^ following RF-PVI and 21%^5^ after cryoballoon PVI. Furthermore, that of the repeat procedure among patients with AF recurrence is approximately 63–95%^3,6,7^. The extra-pulmonary vein (ExtPV) trigger was independently associated with poorer rhythm outcomes following ablation procedures^8^. Consequently, the PVI level, lesion shape, gaps, and extra-PV triggers affect the AFCA rhythm outcome. However, conducting a quantitative evaluation of the anti-AF effects of these conditions remains challenging in patients with AF.

Recent advancements in computational speed have made AF digital twins accessible for clinical research and procedural application^9^. AF digital twins can simulate the results of various virtual interventions under identical and reproducible conditions without posing ethical concerns^10^. Recently, we reported a digital twin study that could evaluate AF wave dynamics and rhythm outcomes by creating PVI gap models^11^. Therefore, we utilized a realistic human AF digital twin, incorporating the left atrial (LA) anatomy and electrophysiology of 50 AF cases.

We hypothesized that conditional alterations in the anatomical PVI levels, lesion widths, gap numbers, and pacing sites for AF induction could influence the anti-AF effects of AFCA. Therefore, this study aimed to compare anti-AF effects quantitatively under varying PVI conditions.

## Results

### Baseline characteristics

Table 1 summarizes the clinical characteristics of the patients. The mean age was 61.3 ± 9.4 years old; 72% were male, and 76% were persistent AF. The mean CHA_2_DS_2_-VASc score was 2.1 ± 1.5, and the presence of the extra PV trigger identified during the isoproterenol provocation protocol was 6% (3/50).

**Table 1:** Baseline characteristics of the patients

Characteristics	
Male, *n* (%, *n*)	72.0% (36/50)
Age (Years)	61.3 ± 9.4
<65, *n* (%, *n*)	58.0% (29/50)
65–74, *n* (%, *n*)	34.0% (17/50)
≥75, *n* (%, *n*)	8% (4/50)
Persistent AF, *n* (%, *n*)	76.0% (38/50)
Duration of AF (Months)	29.4 ± 32.3
BMI (kg/m^2^)	24.4 ± 4.2
CHA_2_DS_2_-VASc score	2.1 ± 1.5
Heart failure, *n* (%, *n*)	28.0% (14/50)
Hypertension, *n* (%, *n*)	48.0% (24/50)
Diabetes, *n* (%, *n*)	16.0% (8/50)
Stroke/TIA, *n* (%, *n*)	22.0% (11/50)
*Echocardiographic parameters*	
LA dimension (mm)	44.0 ± 5.32
LA volume index (mL/m^2^)	43.9 ± 12.4
LVEF (%)	61.5 ± 7.7
E/Em	10.4 ± 3.5
Mean LA voltage (mV)	2.0 ± 0.7
Extra-PV trigger (%)	6% (3/50)

Values are presented as the means ± standard deviations or numbers (percentages).

*AF* atrial fibrillation, *BMI* body mass index, *E* early diastolic trans-mitral flow velocity, *Em* early diastolic mitral annular velocity, *LA* left atrial, *LVEF* Left ventricular ejection fraction, *TIA* transient ischemic attack, *PV* pulmonary vein.

### Overall simulations

**Fig. 1:**
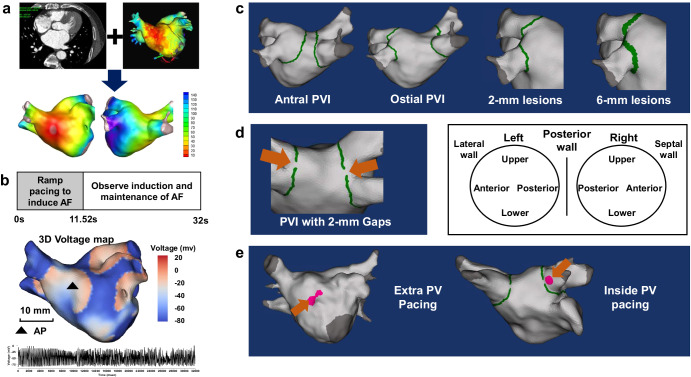
AF digital twins, AF induction protocol, and virtual interventions. **a** We integrated the patient’s CT image and electroanatomical map to create a digital twin of LA. Virtual maps of patients, including voltage, local activation time, fiber orientation, and fibrosis maps, represent LA’s histological and electrophysiological properties. **b** We induced virtual AF with ramp pacing protocol. The action potential of the triangle site shows successful induction and maintenance of virtual AF. **c** We draw the virtual antral line, ostial line with single width (2 mm), and ostial line width triple width (6 mm) for every 50 patients. The green line indicates the ablation line. **d** Orange arrows indicate the gaps of PVI. The standard position of the gap is on both PVs’ upper, lower, anterior, and posterior parts. **e** We changed pacing sites from Bachman’s bundle area to the inside PV. AF atrial fibrillation, AP action potential, CT computed tomography, LA left atrium, PV pulmonary vein, PVI pulmonary vein isolation.

Overall, 600 tests were conducted under varying PVI conditions with 50 patients. Firstly, we performed 50 cases of a basic model simulation where AF induction and maintenance occurred without using other ablation. Next, we drew the antral PVI line and carried out virtual ablation to induce AF in 50 simulations. To compare outcomes based on PVI levels, we created an Ostial PVI model with two types of line width: the existing 2 mm single width and a 6 mm thicker width, each with 50 instances, for 100 simulations (Fig. 1c). To investigate the impact of gaps and the number of gaps, we created 200 cases by adding 1, 2, 4, or 8 gaps (2 mm) to the existing antral PVI model (Fig. 1d). Finally, we generated 200 cases by changing the rapid pacing locations at the extra-PV site (Bachmann’s bundle area) and inside PV with gaps to induce AF (Fig. 1e). These results are summarized in Table 2.

**Table 2:** The total generated models with different conditions from 50 patients

	PVI	PVI level	PVI width	Numbers of gaps	Pacing site	Total numbers
Baseline AF	(−)	(−)	(−)	(−)	(−)	50
Antral PVI	(+)	Antral	(−)	(−)	Extra-PV	50
Ostial PVI (Single width)	(+)	Ostial	Single	(−)	Extra-PV	50
Ostial PVI (Triple width)	(+)	Ostial	Triple	(−)	Extra-PV	50
Gap-1, ExtPVP	(+)	Antral	(−)	1	Extra-PV	50
Gap-2, ExtPVP	(+)	Antral	(−)	2	Extra-PV	50
Gap-4, ExtPVP	(+)	Antral	(−)	4	Extra-PV	50
Gap-8, ExtPVP	(+)	Antral	(−)	8	Extra-PV	50
Gap-1, PVP	(+)	Antral	(−)	1	Inside-PV	50
Gap-2, PVP	(+)	Antral	(−)	2	Inside-PV	50
Gap-4, PVP	(+)	Antral	(−)	4	Inside-PV	50
Gap-8, PVP	(+)	Antral	(−)	8	Inside-PV	50
Overall generated models						600

*AF* atrial fibrillation, *ExtPVP* extra-pulmonary vein pacing, *No.* number, *PV* pulmonary vein, *PVI* pulmonary vein isolation, *PVP* pulmonary vein pacing.

### Effects of PVI level and the lesion width of PVI

Table 3 shows a significant difference in the ratio of final rhythms being AF and final rhythms being AF or AT among the antral PVI, ostial PVI, and baseline AF (*p* < 0.001). The ratio of final rhythms being AF was significantly lower after antral PVI than the ostial PVI. (28% vs.56%, *p* = 0.002). Within the context of ostial PVI, we compared the effects of circumferential linear ablation with a 2-mm width against a circumferential lesion three times wider, simulating single-shot balloon ablation (*n* = 50). The ratio of final rhythms being AF (*p* = 0.840) and final rhythms being AF or AT (*p* = 0.227) did not differ between the ostial ablation lines.

**Table 3:** Rhythm outcomes according to the level and location of PVI

Final rhythm	Antral PVI	Ostial PVI (single width)	Ostial PVI (triple width)	Baseline AF	*P* value
	*N* = 50	*N* = 50	*N* = 50	*N* = 50	
AF, *n* (%)	14 (28.0)	29 (58.0)	27 (54.0)	50 (100)	<0.001
AF or AT, *n* (%)	31 (62.0)	36 (72.0)	42 (84.0)	50 (100)	<0.001

Final rhythm	Antral PVI	Ostial PVI (single and triple width)			*P* value
	*N* = 50	*N* = 100			
AF, *n* (%)	14 (28.0)	56 (56.0)			0.002
AF or AT, *n* (%)	31 (62.0)	78 (78.0)			0.060

Final rhythm		Ostial PVI (single width)	Ostial PVI (triple width)		*P* value
		*N* = 50	*N* = 50		
AF, *n* (%)		29 (58.0)	27 (54.0)		0.840
AF or AT, *n* (%)		36 (72.0)	42 (84.0)		0.227

*AF* atrial fibrillation, *AT* atrial tachycardia, *PVI* pulmonary vein isolation.

### Influences of the number of PVI gaps

**Fig. 2:**
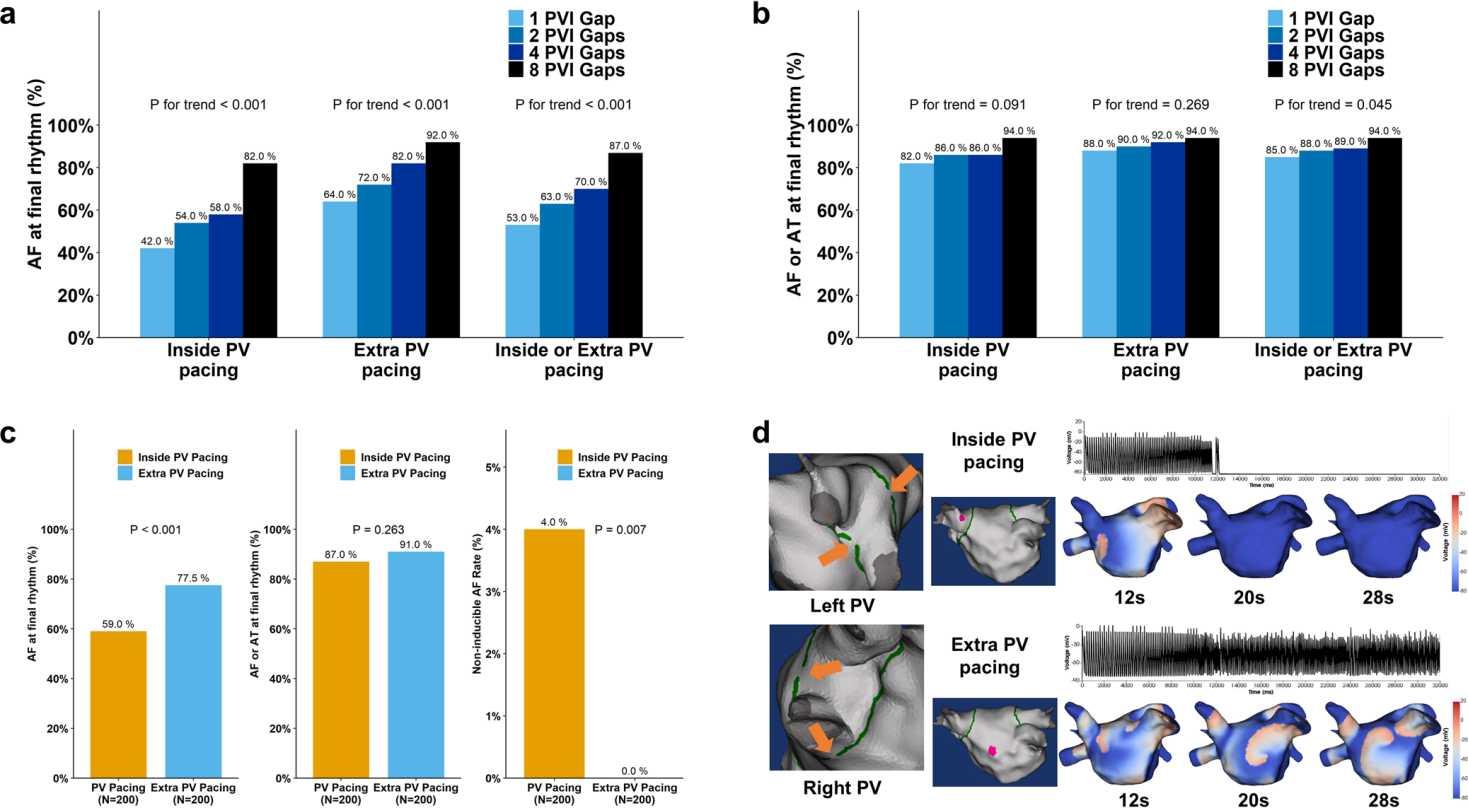
Rhythm outcomes according to the number of PVI gaps and pacing sites. **a** The ratio of AF at the final rhythm according to the numbers of PVI gaps (1 vs. 2 vs. 4 vs. 8). **b** The ratio of AF or AT at the final rhythm according to the numbers of PVI gap (1 vs. 2 vs. 4 vs. 8). **c** Rhythm outcomes including the final rhythm status and non-inducible rate of AF according to pacing site. **d** The orange arrows represent 2 mm gaps on the antral PVI line. The pink dots represent the pacing locations. Both models were created using the same patient, 4 PVI gaps, and locations. However, in the extra-PV pacing model, AF was well sustained. AF atrial fibrillation, AT atrial tachycardia, PV pulmonary vein, PVI pulmonary vein isolation.

Figure 2a and b summarize the anti-AF effects, including the ratio of final rhythms being AF and final rhythms being AF or AT, based on the PVI gap numbers. In both PV pacing models (rapid pacing inside the PV) and extra-PV pacing models (rapid pacing at Bachmann’s bundle area), an increase in PVI gap numbers increased the ratio of final rhythms being AF (*p* for trend < 0.001).

### Extra-PV pacing vs. inside-PV pacing at PVI gaps

The anti-AF effects were compared according to the pacing sites within the PVI gap models (Fig. 2c). The ratio of final rhythms being AF (59.0% in PV pacing vs. 77.5% in extra-PV pacing, *p* < 0.001) significantly increased following extra-PV than inside the PV induction. In comparison, the non-inducible AF rate (4.0% in PV pacing vs. 0% in extra-PV pacing, *p* = 0.007) was significantly reduced. Figure 2d provides a representative example of the impact of pacing location on AF maintenance. The switch in pacing points resulted in distinct outcomes in the same patient, with identical geometry, the same ablation line, and even the same gap numbers and locations. Inside PV pacing showed the disappearance of potential after 12 s. In contrast, the extra-PV pacing model exhibited sustained AF with a pattern of micro-reentry in multiple locations, persisting for up to 32 s.

## Discussion

**Fig. 3:**
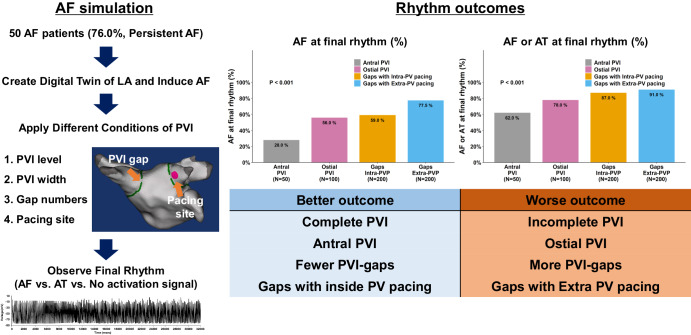
Rhythm outcomes of virtual PVI according to different PVI conditions. Using human AF digital twin technology, we could quantitatively assess and compare the anti-fibrillatory and pro-fibrillatory effects of PVI gaps. Through our simulation experiments, we confirmed the following four key findings. First, wide antral PVI exhibited a significantly more substantial effect on AF termination when compared to ostial PVI. Second, the thickness of the PVI lines did not significantly influence AF maintenance or termination. Third, an increase in the number of PVI gaps correlated with reduced anti-AF efficacy. Lastly, the induction and maintenance of AF were found to be more facile when employing extra PV pacing than intra-PV pacing.

Wide antral circumferential PVI exhibited greater anti-AF effects than ostial PVI but not ablation lesion width. A greater PVI gap occurrence significantly reduced PVI anti-AF effects. With the PVI gaps, rapid extra-PV pacing-induced and maintained AF more easily than inside-PV pacing. Figure 3 summarizes the rhythm outcome sequence. Therefore, atrial mass reduction, PVI gap counts, and pacing sites significantly affected AF maintenance mechanisms. Realistic Human AF computational modeling proves valuable in elucidating AF intervention mechanism, particularly challenging to ascertain clinically or experimentally under reproducible controlled conditions.

Although PVI remains pivotal in AFCA, the anti-AF mechanism in human AF remains unclear^12^. Several theories have attempted to clarify AF maintenance mechanisms, and one of the possible explanations for the AF mechanism is a critical mass theory. Pappone et al.^13^ found a higher ratio of encircled PVI areas in an AF-free group than in an AF-recurrent group. Williams et al.^14^ showed that the mean left atrial effective conducting size was higher in AF-inducible patients than in patients with non-inducible AF. Hwang et al.^15^ observed diminished cardioversion threshold energy, indicating a surrogate marker of critical mass with escalating linear ablations. We previously reported that antral PVI and posterior wall isolation reduced the left atrial critical mass by 17% and 32%^15^, respectively. Therefore, wide antral PVI has better rhythm outcomes than ostial PVI^16^, consistent with our digital twin outcomes.

Regarding the ablation lesion width, the mean radiofrequency PVI width was 3.5–5 mm depending on the ablation index^17^; cryoballoon generates PVI lesions with wider tissue-contact surfaces of 11–15 mm^18^. Our digital twin model showed no difference in anti-AF effects depending on the width of successful PVI lesions, consistent with the clinical rhythm outcome between the radiofrequency and cryoballoon PVIs^19^.

A PVI gap or PV reconnection is a major AF recurrence mechanism following AFCA^20^. During a repeat procedure, the PV reconnection rate ranges from 63% to 95%^3,6,7^. However, PV reconnections exist in patients without AF recurrence^21^. In a randomized clinical trial, Kuck et al.^22^ demonstrated complete PVI superiority over incomplete PVI for AF recurrence within 3 months. This digital twin study showed less effective anti-AF effects with more PVI gaps without ethical issues.

Extra-PV triggers were independently associated with poorer rhythm outcomes post-ablation procedures in clinical studies^8^. We also revealed that the later AF recurrence following catheter ablation, less PV reconnections, and more extra-PV contribute to recurrence during repeat procedures^20^. In this study, the ratio of final rhythm being AF was significantly higher, and non-inducible AF rates were significantly lower after extra-PV induction than in the inside PV induction. It indicated that AF maintenance was more susceptible to extra-PV rapid pacing with PV gaps or PV reconnection than intra-PV rapid pacing.

We could not only provide tailored therapies to each patient but also make better clinical interpretability by using the digital twin technology^23^. We have developed our digital twin technology by incorporating the images of the computed tomography (CT) and electroanatomical map (EAM) obtained during the procedure. Our current digital twin model program (CUVIA) has been developed based on bipolar electrogram (EGM) data, utilizing a standardized mapping system for a long time. We have continuously improved and evolved the program, gaining experience for its application in real-time ablation strategies through several randomized controlled trials, which were published^9,24–26^ or are still ongoing. To enhance the development of a robust model, new mapping systems such as high-density mapping or noncontact dipole density mapping system^27^ (Acutus Medical, Carlsbad, CA) might have certain advantages over contact mapping systems, as it allows obtaining high-resolution data points throughout the entire left atrium. Furthermore, in this study, we set the pacing site to a single location and compared outcomes between two pacing positions. However, Zahid et al.^28^ identified atrial fibrosis using multiple pacing sites, and Boyle et al.^29^ demonstrated the effectiveness of pacing around fibrotic areas to locate re-entrant drivers. This suggests that in our model, employing multiple pacing sites or pacing around fibrotic regions could yield meaningful insights, such as differences in rhythm outcomes or the identification of rotors or re-entrant drivers. However, we kept a consistent ramp pacing protocol on the earliest LA activation, mimicking the clinical AF induction by high right atrial pacing.

This study had some limitations. First, the simulations were performed using the mathematical model using one set of AF settings for all patients. In reality, it is not proven that all the patients would follow this simplified model and settings. However, to generate a more accurate model, we provided the virtual fibrosis, fiber orientation, and local activation maps with different electrical properties based on the clinical EAM. Second, our model only simulates LA. Although we could provide a bi-atrial model^30^, we thought that the left atrium model was enough to prove the alterations of the PVI and the gap of PVI. Third, though we used bipolar voltage signals, these data had poor reproducibility and were dependent on various factors such as directionality and conduction velocity. As mentioned earlier, fiber orientation was implemented based on the atlas, and conduction velocity was adjusted according to the propagation direction. However, it is acknowledged that these manually drawn orientations may not precisely match those of actual patient fibers, indicating a limitation. Additionally, due to the impossibility of mapping all LA points, the use of the inverse distance weighting (IDW) method could introduce errors, posing another limitation. Fourth, certainly, evaluating atrial fibrosis through bipolar voltage measurement is influenced by factors^31^ such as activation vector, angle of incidence, electrode size^32^, interelectrode spacing, tissue contact, filtering, mapping density, reproducibility, and others. We previously reported^33^ the influence of catheter orientation, catheter contact angle, local conduction velocity, scar size, and catheter type on the bipolar electrogram morphology using digital twin technology. Increased catheter orientation, increased contact angle, decreased conduction velocity, AF conditions, and increased scar size were associated with reduced bipolar voltage. Otherwise, higher voltages were obtained by narrowly spaced and smaller electrodes. Thus, this would be one of our limitations. However, to overcome the sensibility and disadvantages of bipolar voltage maps, we might use unipolar noncontact mapping to obtain more accurate information. Fifth, there were several theories to clarify AF maintenance mechanisms, including arrhythmogenic Purkinje-like cells within PV myocardial sleeves^34^ or ganglionated plexi around the PV antrum^35^. However, we did not include this information in our digital twin model. Sixth, our digital twin model was composed of a monolayer without transmural. Lastly, although patients were mainly persistent atrial fibrillation, the proportion of the presence of extra PV triggers was relatively low (6%). It was difficult to compare the influence of real extra PV triggers in the virtual setting.

We observed reduced anti-AF effects for PVI in the following order: antral PVI, ostial PVI, inside-PV pace-induction with gaps, and extra-PV pace-induction with PV gaps. A realistic digital twin of human AF is valuable in elucidating the AF intervention mechanism, which is challenging to substantiate clinically or experimentally under reproducibly controlled conditions.

## Methods

**Fig. 4:**
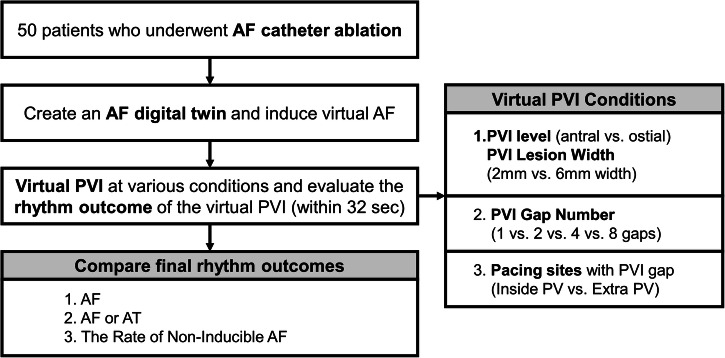
Flow chart of the study. We included 50 AF patients from the Yonsei AF ablation cohort. We generated digital twins of LA and induced virtual AF, and compared rhythm outcomes according to different virtual interventions. Different conditions include PVI level and width, number of PVI gaps, and pacing location. Rhythm outcomes include final rhythm status and the rate of non-inducible AF. AF atrial fibrillation, AT atrial tachycardia, LA left atrium, PV pulmonary vein, PVI pulmonary vein isolation.

In our previously published articles, we described LA modeling and AF digital twins^10,36,37^. To create the digital twin, we utilized the modified human atrial myocyte model, the so-called Courtemanche–Ramirez–Nattel model^38^. We also developed a GUI software (CUVIA, Model: SH01; Laonmed Inc., Seoul, Korea) capable of generating a realistic three-dimensional (3D) in silico digital twin and performing virtual interventions using patient-specific data. Figure 4 shows the overview of our study, and Fig. 1 shows the summarized computational modeling process utilized in this study.

### Study population

The Institutional Review Board of Severance Cardiovascular Hospital, Yonsei University Health System, approved this study. The study was performed in accordance with the principles of the Declaration of Helsinki. All patients in the Yonsei AF Ablation Cohort Database (ClinicalTrials.gov Identifier: NCT02138695) provided written informed consent for their clinical data to be used in digital twin studies. In this study, 50 patients with AF who underwent AFCA were enrolled. The inclusion criteria were as follows: (i) age ≥19 years, (ii) AAD resistance, and (iii) availability of 3D electroanatomical map-integrated computed tomography (CT) images. The exclusion criteria were as follows: (i) significant structural heart disease, (ii) valvular AF, and (iii) ablation or cardiac surgery history.

### 3D-digital twin of the LA

To generate a digital twin of the LA for each patient, several sequential steps were performed. First, 3D meshes comprising triangular nodes were developed based on individual LA CT images. This 3D mesh surface consisted of ~400,000–500,000 triangular nodes, with a mean spacing of 235.1 ± 32.1 μm between adjacent nodes. Second, to obtain the virtual 3D mesh electrical and histological properties, clinical electroanatomical maps (EAM) were acquired. Throughout the AFCA procedure, we collected bipolar electrogram data from more than 500 points on the LA surface using a circular mapping catheter and an Ensite NavX system (Abbott Inc., Chicago, IL, USA) during a paced rhythm with a cycle length of 500 ms. Subsequently, we aligned the coordinates of the electroanatomical map with individual CT images, producing a clinical EAM. Third, the process of aligning the clinical EAM with 3D mesh models consists of four steps: (1) geometry, (2) trimming, (3) field scaling, and (4) alignment. In the geometry step, an EAM was created, as mentioned earlier. During the trimming phase, artifacts arising due to the breathing of the patients were removed, and the PV and LA appendage (LAA) sites were demarcated based on the ostial positioning. During the field scaling stage, the electroanatomical map was optimized according to the interelectrode spacing to ensure the closest match between the sizes of the 3D meshes and that of the CT image. Finally, by accurately positioning alignment points at the defined ostium, the process of registering the clinical EAM onto the mesh was successfully concluded.

Subsequently, we employed clinical voltage map interpolation based on the inverse distance weighting method to create the virtual voltage map^39^. Following a precise aligning of the voltage and CT image data on the 3D LA model via rotation and translation, we interpolated a clinical voltage map to produce a virtual voltage map on the 3D model. We then developed fiber orientations utilizing the atlas-based mesh method for tracking and visualization^40^. We achieved fiber orientation maps by directly drawing the fiber orientation map onto a 3D model created based on the CT images of the patient. Representative vectors were manually drawn, and fiber orientations at surrounding nodes were determined through interpolation around these representative vectors. A vector aligned with the myocardial fiber direction was generated at each point in the heart. The conductivity in the direction orthogonal to the vector was lower than the conductivity along the vector direction.

A fibrosis map of 3D meshes was obtained from the interpolated voltage data. We used the equation for the probability of fibrosis and bipolar voltage^33^. The conduction velocity (CV) and ionic currents of nodes exhibited variation contingent upon the fibrosis state of each node. Compared with normal cells, the ion current of the fibrosis cell, that is, the inward rectifier potassium current (*I*_K1_), L-type calcium current (*I*_CaL_), and sodium current (*I*_Na_) decreased by 50%, 50%, and 40%, respectively^28^.

Once the fiber orientation map and fibrosis map were generated, we determined the electrophysiological parameters of fibrotic and non-fibrotic tissue. We defined the longitudinal conduction velocity as that in the same direction as the vector and the transversal conduction velocity as that in the perpendicular direction to the vector. The conductivity of the model was applied at 0.1264 S/m (non-fibrotic longitudinal cell), 0.0546 S/m (fibrotic longitudinal cell), 0.0252 S/m (non-fibrotic transverse cell), and 0.0068 S/m (fibrotic transverse cell)^28^.

We synchronized the clinical and virtual local activation time (LAT) map to determine CV. Using the CUVIA program, the clinical local activation time map was displayed on the screen of the program, and an experienced researcher synchronized a virtual local activation time map to match the appearance of the virtual map with the clinical one. By matching the CV of the virtual LAT map with that of the clinical LAT map, we adjusted the diffusion coefficients of the 3D model.

### Virtual AF induction

Ionic currents during sinus rhythm or AF in each cell were determined using the human atrial myocyte model established by Courtemanche et al.^38^. All ion currents were set to 100% during the sinus rhythm phase. Before inducing virtual AF, the ion channel currents were modified to represent the remodeling of ion currents seen during AF. Specifically, the sodium current (*I*_Na_), transient outward potassium current (*I*_to_), L-type calcium current (*I*_CaL_), ultra-rapid outward current (*I*_Kur_), and calcium current concentration in the uptake compartment (*I*_Caup_) decreased by 10%, 70%, 70%, 50%, and 20%, respectively. The inwardly rectifying potassium current (*I*_K1_) was elevated by 110% compared to SR^41^. Supplementary Table 1 provides comprehensive information regarding the ionic current setting. To induce virtual AF, we ramped pacing around Bachman’s bundle for 11.52 s with eight beats per cycle. The pacing started at 200 ms and decreased by 10 ms intervals until it reached 120 ms. Furthermore, we placed them inside the PV gaps to assess the final rhythm. We observed whether the virtual AF persisted for 32 s, including the pacing time. All virtual interventions, including PVI, PVI gaps, or different pacing sites for AF induction, were performed before ramp pacing (0 s).

### Virtual PV intervention protocols

Using a CUVIA digital twin (Laonmed Inc., Seoul, Korea), circular lesions of 2 mm width were created in both side PVs. We conducted PVI at the antral and ostial levels to compare ablation location effects (Fig. 1c). The PV ostia were lines connecting the saddle regions at the PV-LA junction of the upper PV (rooftop or lower PV bottom) and PV carina. The PV antrum was defined by connecting a straight line from top to bottom along the posterior side of the LA at the maximal inflection point of the round surface of the LA exterior, adjacent to the PV ostium^42^. To evaluate the effect of the PVI-lesion width, we compared the 2-mm width ostial PVI and 6-mm width ostial PVI (three times wider) while maintaining the same PV-LA margin of PVI (Fig. 1c)^43^.

To investigate the role of the number of PVI gaps on the anti-AF effects, we established 2 mm-sized gaps capable of generating wave breaks^44^ along the PVI lines (Fig. 1d). The number and location of these gaps were assigned randomly to the bilateral PVs in the AF model of 50 patients: 50 PVI-1 gaps, 50 PVI-2 gaps, 50 PVI-4 gaps, and 50 PVI-8 gaps. We defined the “PVI gaps” at eight different sites (top, bottom, anterior, or posterior sides of the PV): eight combinations with a single PVI gap, 28 combinations with two gaps, 70 combinations with four gaps, and a combination with eight PVI gaps in the digital twin of each patient. Using randomization, we chose one representative model for each number of PVI gaps from the AF modeling of the 50 patients. Overall, 600 digital twin models were analyzed with or without PVI gaps (Table 2).

### Different rapid-pacing sites at the PV or extra-PV with PVI gaps

Under the complete PVI condition, electrical conduction from the PV to the LA, or vice versa, was blocked bidirectionally in the digital twins. However, in cases where PVI gaps existed due to an incomplete bidirectional block, rapid PV and LA pacing can induce AF. Therefore, we compared the pro-fibrillatory effects of rapid pacing from Bachman’s bundle area and inside the PV under PVI gap conditions, simulating the extra-PV of PV triggers (Fig. 1e).

### Evaluation of rhythm outcomes

**Fig. 5:**
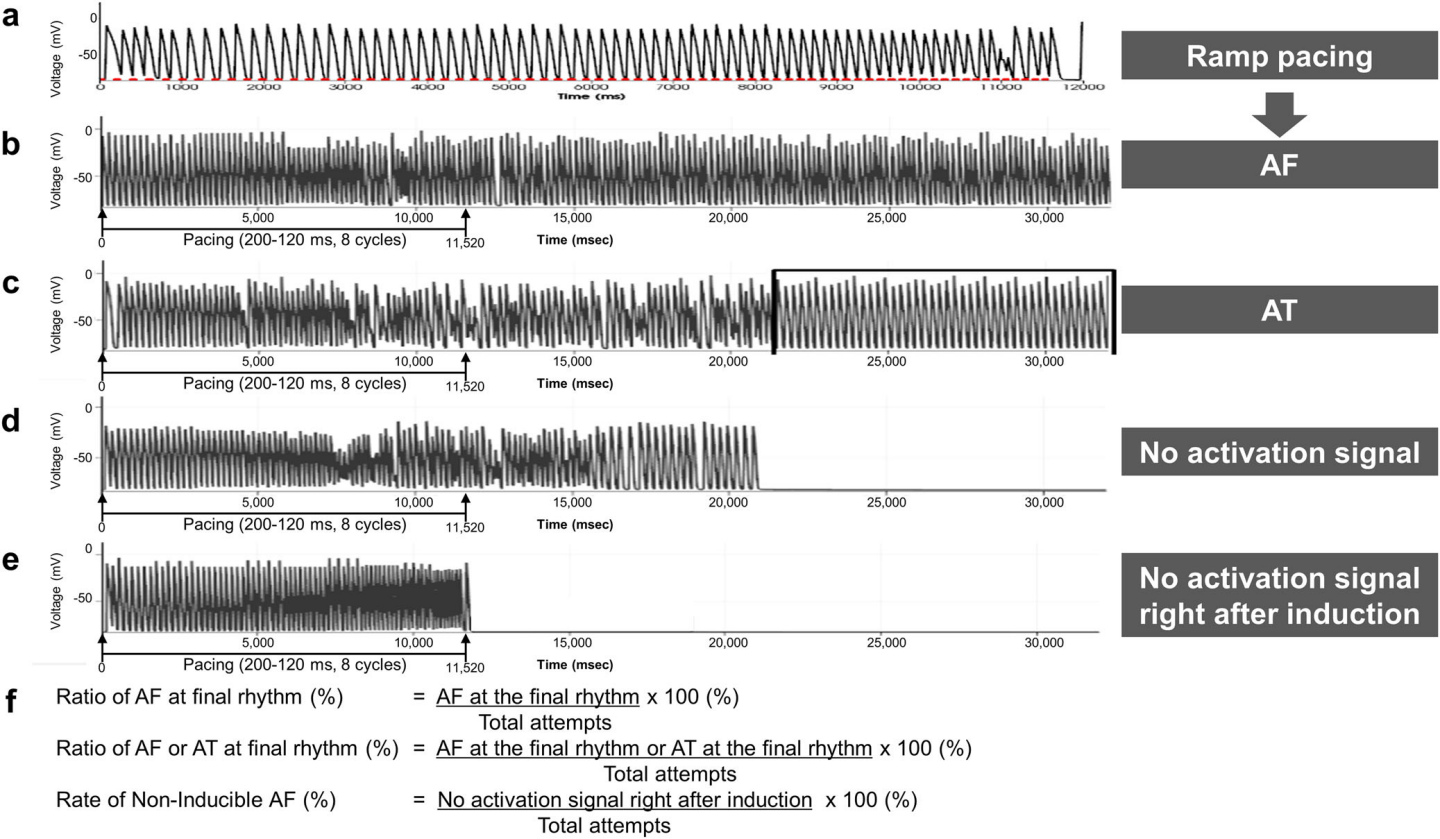
Possible rhythm outcomes and calculation methods after induction of virtual AF. **a** The pacing started at 200 ms and decreased by 10 ms intervals until it reached 120 ms to induce AF. Red dots indicate pacing stimulation markers. **b** Induced AF remained after 32 s. **c** AF converted to regular AT at 22 s. **d** No activation signal was observed after 21 s. **e** No activation signal was observed right after 11.52 s of induction protocol. **f** The calculation methods of rhythm outcomes. AF atrial fibrillation, AT atrial tachycardia, PV pulmonary vein.

At 32 s following AF induction in the AF digital twin, the resulting rhythm outcomes were categorized as follows: AF maintenance, conversion to regular atrial tachycardia (AT), and no activation signal (Fig. 5). No activation signal would be further separated according to the timing of inactivation.

- *Failure to induce AF* was defined as the absence of an activation signal at the final observation moment (32 s), especially right after 11.52 s of pacing induction protocol.
- *The non-inducible AF rate* was calculated as the ratio of failure to induce AF to the total number of induction attempts.

We compared rhythm outcomes according to different PVI conditions, such as the level of PVI, PVI width, the number of PVI gaps, and the location of the pacing site.

### Statistical analysis

Categorical variables were presented as numbers (percentages). To assess normal distribution, continuous variables were analyzed using Shapiro–Wilk or Kolmogorov–Smirnov tests. Continuous variables without a normal distribution were expressed as medians with interquartile ranges, whereas variables with a normal distribution were expressed as means ± standard deviation. The proportions of categorical variables were compared among the groups using the Chi-square or Fisher’s exact tests. Continuous variables without a normal distribution were analyzed using the Mann–Whitney *U* test for two-group comparisons and the Kruskal–Wallis test for three-group comparisons. Continuous variables with a normal distribution were examined using a *t*-test for a two-group comparison and an analysis of variance test to compare the three groups. Spearman’s correlation was used to analyze trends in continuous variables. The Cochran–Armitage test for the trend was used to analyze trends within the categorical variables. Statistical significance was defined as a two-sided *P*-value of <0.05. All statistical analyses were performed using R version 3.6.0 (R Foundation for Statistical Computing, Boston, MA, USA).

### Supplementary information

Supplementary information

## Data Availability

The data collected and analyzed in this study are only available from the corresponding author upon reasonable request and with permission of the institution review board.

## Code availability

Analyses were performed in R (v3.6.0). The underlying code for this study is available from the corresponding author upon reasonable request.
