# Supplementary material for: Anti- and pro-fibrillatory effects of pulmonary vein isolation gaps in human atrial fibrillation digital twins
Source: NPJ Digit Med. 2024 Mar 26;7:81. doi: 10.1038/s41746-024-01075-y (PMC10966060; doi:10.1038/s41746-024-01075-y)
Supplement: Supplementary file 1 — Supplementary information [file 41746_2024_1075_MOESM1_ESM.pdf]

Supplementary Table 1 The ion current settings of sinus rhythm and AF

	SR (%[1])	AF (%[1])
I_{Na}	100	90
I_{K1}	100	210
I_{to}	100	30
I_{Kr}	100	100
I_{CaL}	100	30
I_{Kur}	100	50
I_{Ks}	100	100
$Ca_{up(Max)}$	100	80
I_{KAch}	100	100

AF, atrial fibrillation; I_{Na}, Fast inward sodium current; I_{K1}, Inward rectifier potassium current; I_{to}, Transient outward potassium current; I_{Kr}, Rapid delayed rectifier potassium current; I_{CaL}, L-type inward calcium current; I_{Kur}, Ultrarapid delayed rectifier potassium current; IKs, Slow delayed rectifier potassium current; $Ca_{up(Max)}$, Maximal calcium current concentration in the uptake compartment; I_{KAch}, Acetylcholine activated potassium current; SR, sinus rhythm; Values are presented as numbers (percentages).

[1] Maximal conductance of current of each ion channel.
